# Co-circulation of two sublineages of HPAI H5N1 virus in the Kingdom of Saudi Arabia with unique molecular signatures suggesting separate introductions into the commercial poultry and falconry sectors

**DOI:** 10.1099/vir.0.2008/004259-0

**Published:** 2008-11

**Authors:** Isabella Monne, Alice Fusaro, Mohamed Hamad Al-Blowi, Mahmoud Moussa Ismail, Owais Ahmed Khan, Gwenaëlle Dauphin, Astrid Tripodi, Annalisa Salviato, Stefano Marangon, Ilaria Capua, Giovanni Cattoli

**Affiliations:** 1 Istituto Zooprofilattico Sperimentale delle Venezie, OIE/FAO and National Reference Laboratory for Newcastle Disease and Avian Influenza, Viale dell'Università 10, Legnaro, Padova, Italy; 2 Veterinary Labs Administration, Ministry of Agriculture, Riyadh, Kingdom of Saudi Arabia; 3 Department of Poultry Diseases, College of Veterinary Medicine, Kafr-Elsheikh University, Egypt; 4 Central Veterinary Diagnostic Laboratory, PO Box 15831, Riyadh 11454, Kingdom of Saudi Arabia; 5 Animal Health Service, Food and Agriculture Organization of the United Nations, Viale delle Terme di Caracalla, 00153 Rome, Italy

## Abstract

Since early 2007, the Kingdom of Saudi Arabia (KSA) has experienced several highly pathogenic avian influenza (HPAI) H5N1 outbreaks in the falconry and poultry sectors. The public health threat associated with peculiar husbandry systems, requiring close contact between humans and birds of prey, highlights the need of an improved understanding of the epidemiology and of the viral characteristics of H5N1 viruses circulating in the region. Here we report molecular and phylogenetic analyses of H5N1 viruses isolated in the KSA in 2007 in distinct compartments of avian husbandry. From the results of our investigation it appears that two separate introductions into the different sectors occurred. The identification of specific amino acid mutations, which are described as genetic signatures of human influenza A viruses or known to confer resistance to antiviral drugs, raises concerns for the possible human health implications of the KSA H5N1 viruses.

Since late 2005, highly pathogenic avian influenza (HPAI) viruses of H5N1 subtype have spread into Europe, the Middle East and Africa affecting both wild birds and poultry (Alexander, 2007; Salzberg *et al.*, 2007). Three distinct sublineages were identified in these regions, termed EMA1, EMA2 and EMA3 by Salzberg *et al.* (2007), but designated 2.2.1, 2.2.2 and 2.2.3 in this manuscript, respectively. Incursions into the European Union have been seen mainly in wild birds, and the limited outbreaks occurring in poultry, as a result of the spread from wild birds or from poultry to other domestic birds, have been promptly eradicated in the vast majority of the European countries (Pittman & Laddomada, 2008). In contrast, in Africa and in the Middle East, since the first outbreaks reported in early 2006, there has been extensive spread and circulation of H5N1 virus in poultry, but only an extremely limited number of isolations from wild birds (Fasina *et al.*, 2007; Gaidet *et al.*, 2007). To date it is still unclear how H5N1 virus reached these two regions, whether through wild bird movements, trade of infected poultry commodities or both.

Between February and April 2006, infections due to H5N1 HPAI virus were reported in five Middle Eastern countries, namely: Iraq and Kuwait (February 2006); Jordan and Israel (March 2006) and Palestine (April 2006). In the same period, one suspected H5N1 case was reported in falcons in the Kingdom of Saudi Arabia (KSA) (ProMED-mail, 2006). However, the first confirmed case of HPAI H5N1 in KSA was dated 31 March 2007 (OIE, 2008). Since then, KSA experienced a number of outbreaks (*n*=29, as of 15 April 2008) (OIE, 2008) of HPAI H5N1 in three distinct compartments: falconry, backyard poultry and, at a later stage, industrially reared poultry. Initially, the reported outbreaks were limited to the backyard poultry and falconry sectors. But, on 14 November 2007, the first outbreak in the commercial sector was reported in a broiler breeder flock in the Al Kharj region of KSA.

The first case of H5N1 infection in KSA was reported from specimens collected from imported Houbara bustards (*Chlamidotis undulata macqueenii*) (H. Aidaros, A. Tripodi and N. Honhold, FAO/OIE Crisis Management Centre, Rome, personal communication). These birds are a traditional, highly prized quarry of falconers and they are traded extensively from Central Asia to the Middle East (Bailey *et al.*, 2000). The art of falconry is an important cultural activity in the Arabian Peninsula which has been practised for more than a millennium. Falcons are often raised in households and handled on a daily basis by owners and caretakers. The falconry activities involve both keeping birds of prey for hunting and rearing other birds, such as Houbara bustards, which are used as prey. It has been estimated that the illegal trade of falcons from Central Asia to the Middle East may involve as many as 14 000 or more birds annually (ProMED-mail, 2006). Thus, trading of live birds (both predators and prey) associated with falconry represents a potential vehicle for introduction and spread of avian influenza viruses in the KSA and in other countries of the Gulf area. In addition, falcon husbandry methods may result in an increased risk of human exposure to H5N1, compared with other avian rearing practices. An improved understanding of the epidemiology and of the viral characteristics of H5N1 viruses circulating in the region would appear essential in managing the animal and human health risks.

In this paper we report the first molecular and phylogenetic analyses of H5N1 viruses isolated from the Kingdom of Saudi Arabia (KSA) from distinct compartments of avian husbandry.

Following standard virus isolation procedures (OIE, 2005) at the Central Veterinary Laboratory in Riyadh (KSA), 20 viruses were isolated from 20 different outbreaks and sent to the OIE/FAO Reference laboratory in Italy (IZSVe) for confirmatory diagnosis and molecular analysis (Table 1) as part of the FAO assistance mission in the country. Samples were processed for subtyping and pathotyping as described previously (Alexander & Spackman, 1981; European Commission, 2006). Subsequently, based on the results obtained from analysis of the 20 haemagglutinin (HA) gene sequences, the whole genome of each of eight representative isolates was sequenced (Table 1).

The amplification of the eight viral gene segments was done by RT-PCR using gene-specific primers (available upon request). PCR products were purified (ExoSAP-IT) and sequenced in a 3130xl Genetic Analyzer (Applied Biosystems). Phylogenetic analysis was performed using the neighbour-joining method in the mega3 programme (Kumar *et al.*, 2004). The tree topology was confirmed by the generation of a maximum-likelihood (ML) tree estimated using dnaml in the phylip v3.6 package (tree not shown) (Felsenstein, 1989). The GenBank/EMBL/DDBJ accession numbers of the eight gene segments of the isolates A/Houbara bustard/Saudi Arabia/6732-1/2007 and A/turkey/Saudi Arabia/6732-6/2007 are EU445682, EU590684–EU590695, EU424135, EU424136 and EU596411.

Phylogenetic analysis of the HA gene revealed that all the 20 KSA isolates belonged to genetic clade 2.2 (WHO/OIE/FAO H5N1 Evolution working group, 2008) and that they were closely related to the viruses circulating in birds throughout Europe, Russia, Africa and the Middle East since late 2005.

Interestingly, the phylogenetic trees produced for each of the eight genes showed that the sequences of the KSA viruses grouped into two distinct sublineages.

The nucleotide (nt) sequences of the entire genome of the viruses isolated from domestic birds (A/ostrich/Saudi Arabia/6732-3/2007, A/turkey/Saudi Arabia/6732-6/2007, A/duck/Saudi Arabia/6732-7/2007, A/chicken/Saudi Arabia/6732-13/2007, A/chicken/Saudi Arabia/6732-4/2007, A/chicken/Saudi Arabia/6732-18/2007) were closely related to each other for all eight gene segments and were placed in sublineage 2.2.2 (Fig. 1). The highest similarity was seen with H5N1 viruses of the same sublineage isolated in Nigeria during 2006 (the nt identity for the HA gene ranged between 98.9 and 99.6 % with the A/ck/Nigeria/FA6/2006 strain). In contrast, the two viruses isolated from the Houbara bustard and falcon that were studied (A/Houbara bustard/Saudi Arabia/6732-1/2007 and A/falcon/Saudi Arabia/6732-2/2007) clearly clustered in sublineage 2.2.3. Interestingly, these two viruses had highest nt similarity in the HA gene with H5N1 viruses isolated in Kuwait from domestic birds and a falcon (99.8 % similarity with strain A/falcon/Kuwait/1019-7/2007) (Fig. 1).

At the amino acid level, the HA0 cleavage site sequence of all 20 isolates was PQGERRRKKR*GLF, which is identical to those of recent African, Asian and European H5N1 HPAI strains.

Amino acid sequence analysis of the surface proteins revealed that KSA isolates did not possess mutations linked to an increased affinity toward human-like sialic acid substrates in the binding domain of the HA protein (Matrosovich *et al.*, 1997). No molecular changes associated with a modified susceptibility to neuraminidase inhibitors were observed in the neuraminidase protein of the strains analysed. Notably, sequence analysis of the M2 ion channel protein showed that the KSA viruses belonging to 2.2.2 sublineage had the amino acid substitution S31N. It has been reported that this mutation confers resistance to adamantanes, a group of antiviral drugs used for treatment and prevention of human influenza A virus infections (Ilyushina *et al.*, 2005; Suzuki *et al.*, 2003). This substitution has been detected in amantadine-resistant H5N1 influenza viruses isolated from humans and poultry in Asia between 1996 and 2005 (Cheung *et al.*, 2006; Ilyushina *et al.*, 2005).

The E627K mutation in the PB2 gene, which has been associated with an increase of virulence of influenza A viruses for mammals (Subbarao *et al.*, 1993), was also detected in all the KSA viruses analysed in the present study.

The amino acid sequence analyses of internal genes revealed the existence of three other host-specific mutations. In the NS1 gene of all the KSA strains belonging to the 2.2.2 sublineage, we observed the E227K mutation (Table 2). This mutation is located in the extreme C-terminal of the NS1, which has been demonstrated to modulate pathogenicity of avian influenza (AI) through mechanisms not completely clarified yet (Jackson *et al.*, 2008; Obenauer *et al.*, 2006). In particular the four C-terminal residues of the gene correspond to a PDZ ligand domain, which is a protein–protein recognition module that organizes diverse cell-signalling assemblies (Sheng & Sala, 2001). Avian influenza viruses naturally possess glutamic acid (E) at position 227, while human influenza viruses contain arginine (R) (Chen *et al.*, 2006). Finkelstein *et al.* (2007) observed that H1N1 subtype viruses circulating during the 1918 ‘Spanish’ human pandemic, which are thought to have an avian progenitor virus, possessed the amino acid lysine (K) in this position, and were therefore similar to the KSA strains analysed in the present study. A recent reverse genetics study indicated that recombinant viruses containing C-terminal NS1 sequences from the 1918 H1N1 and some recent H5N1 HPAI viruses show increased virulence in mice (Jackson *et al.*, 2008). The results of the blast search showed that the KSA viruses are the first reported H5N1 strains of the Qinghai (2.2) lineage possessing this mutation and apart from these, it seems that the E227K mutation has only been recorded in two H5N1 Indonesian strains isolated in 2005 (GenBank accession numbers CY014189 and CY014196). Thus, the E227K mutation appears to be a rare amino acid signature which in this case was presumably acquired by H5N1 viruses in KSA in 2007.

Host markers conserved in the human influenza virus population (Chen *et al.*, 2006) were detected in the PB1 gene (R327K) of the 2.2.2 lineage KSA strains and in the NP gene (V33I) of A/Houbara bustard/Saudi Arabia/6732-1/2007, A/falcon/Saudi Arabia/6732-2/2007, A/ostrich/Saudi Arabia/6732-3/2007 and A/chicken/Saudi Arabia/6732-4/2007 strains (Table 2).

From the results of the present investigation it appears that two distinct sublineages (2.2.2 and 2.2.3) are co-circulating in KSA in different compartments of avian husbandry. It can be inferred from this that there were two separate introductions of H5N1 into KSA, and that despite the circulation of H5N1 for almost a year, there appears to have been no spill-over from the captive bird to the domestic bird population or vice versa, suggesting that the two compartments are epidemiologically separated. This is apparently in contrast to the situation in neighbouring Kuwait, where 2.2.3 viruses apparently circulate in both populations (Fig. 1).

The strains isolated from poultry in KSA were closely related to poultry viruses isolated in Nigeria. Surprisingly, the recent 2007 KSA poultry isolates clustered together with early Nigerian strains obtained in 2006, rather than with viruses that appeared to be predominant in 2007 (Monne *et al.*, 2008). This suggests an early epidemiological connection between KSA and Nigeria resulting in spread from one country to another or that the viruses spreading to these two countries came from a common source. Spread of descendants of early Nigerian viruses seems to have occurred efficiently in diverse avian species reared commercially (chickens, turkeys, ostriches and ducks) and farms located in different parts of the Kingdom, suggesting poor biosecurity across the commercial sector.

Our findings in KSA do not represent the first report of multiple introductions of HPAI H5N1 avian influenza viruses into a given country. The three distinct H5N1 sublineages were identified in wild birds in Germany between 2006 and 2007 (Starick *et al.*, 2008). During 2006, two distinct sublineages of the HPAI H5N1 were also introduced into Italy (Salzberg *et al.*, 2007) and France (Le Gall-Reculé *et al.*, 2008) through wild birds.

To the best of our knowledge, the introduction of multiple H5N1 genogroups in European countries has not resulted in the generation of reassortant strains. This is probably due to the fact that infections in Europe have been mainly confined to wild bird populations and extensive virus circulation in poultry has been avoided.

In contrast, it was shown recently that the co-circulation of two sublineages (2.2.1 and 2.2.2) in Nigerian poultry resulted in the generation of a novel 2.2.1/ 2.2.2 reassortant strain that became predominant in 2007 (Monne *et al.*, 2008). Based on the results of the present study, it is possible that a novel scenario exists in the KSA. In this country, multiple introductions of HPAI H5N1 viruses have led to the co-circulation of at least two different virus sublineages, which appear so far to have been maintained separated in distinct avian compartments. However, this separation might not be maintained in the future, especially if spread of the virus is not efficiently controlled in the country. Control policies may be aided by vaccination of susceptible birds, including falcons (Lierz *et al.*, 2007).

In addition to the virulence markers that are present in most Qinghai-lineage (2.2) viruses, such as the PB2 E627K mutation, the KSA isolates of 2.2.2 sublineage from domestic birds exhibited a specific mutation, S31N, in the M2 protein, which is associated with resistance to adamantane antivirals such as amantadine. Other mutations, known as genetic markers of human influenza A viruses, namely NS E227K, PB1 R327K and NP V33I, have also been detected (Table 2). These findings raise concerns for the possible human health implications of these genotypes, in view of the local husbandry methods.

It should be noted that in the Middle East, in addition to the circulation of the H5N1 viruses described here, there is evidence of extensive circulation of H9N2 viruses (Aamir *et al.*, 2007, Monne *et al.*, 2007). The co-circulation of H9N2 and H5N1 subtypes of AI increases concerns about the generation of reassortant viruses with potential animal and human health implications.

The findings of this study indicate that improved surveillance programmes are essential to monitor the situation in the KSA.

## Figures and Tables

**Fig. 1. f1:**
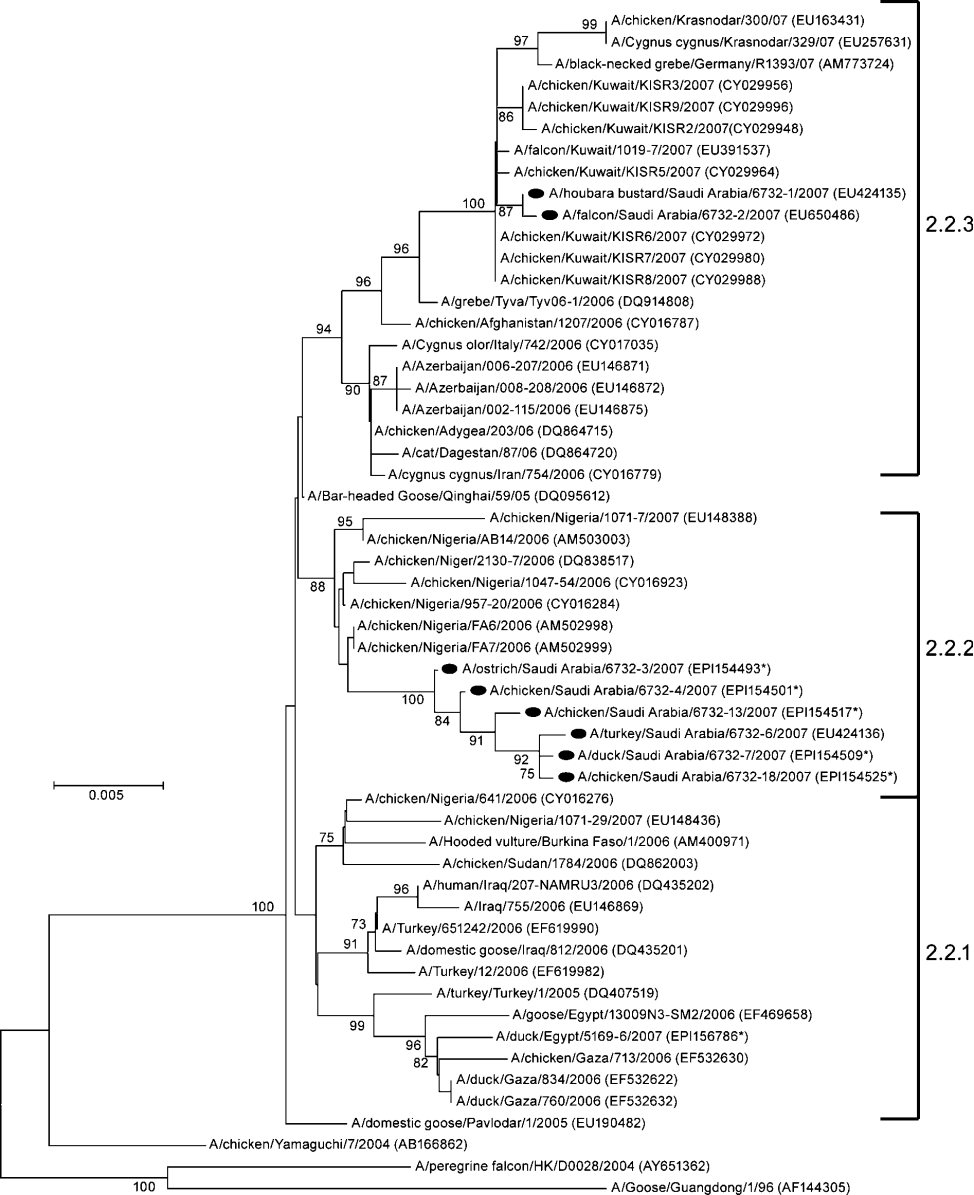
Phylogenetic tree for the HA gene constructed by the neighbour-joining method, which includes eight representative KSA viruses. The evolutionary distances were computed using the maximum composite likelihood method and are in the units of the number of base substitutions per site. Sequences obtained in this study were labelled with a filled circle. The numbers at each branch point represent bootstrap values and were determined by bootstrap analysis using 1000 replications. Bar, 0.005 nucleotide substitutions/site; *, sequence available in the EpiFlu database at the Global Initiative on Sharing Avian Influenza Data (GISAID; http://platform.gisaid.org/).

**Table 1. t1:**
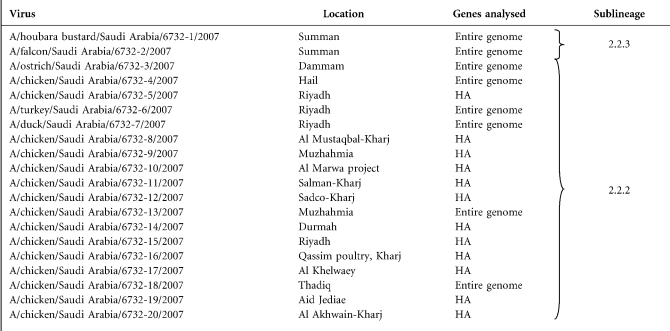
List of H5N1 influenza viruses analysed in the present study

**Table 2. t2:** Typical amino acid signature of human influenza viruses observed in the Arabian strains

**Protein**	**Position (aa)**	**Predicted aa**		**Viruses with detected mutations**	**Mutation**	**References**
		**Avian**	**Human**			
PB2	627	E	K	All strains analysed in the present study	E627K	Subbarao *et al.* (1993); Chen *et al.* (2006)
NS1	227	E	R or K (H1N1 1918)	A/ostrich/Saudi Arabia/6732-3/2007A/chicken/Saudi Arabia/6732-4/2007A/turkey/Saudi Arabia/6732-6/2007A/duck/Saudi Arabia/6732-7/2007A/chicken/Saudi Arabia/6732-13/2007A/chicken/Saudi Arabia/6732-18/2007	E227K	Chen *et al.* (2006); Finkelstein et al. (2007)
PB1	327	R	K	A/ostrich/Saudi Arabia/6732-3/2007A/chicken/Saudi Arabia/6732-4/2007A/turkey/Saudi Arabia/6732-6/2007A/duck/Saudi Arabia/6732-7/2007A/chicken/Saudi Arabia/6732-13/2007A/chicken/Saudi Arabia/6732-18/2007	R327K	Chen *et al.* (2006)
NP	33	V	I	A/houbara bustard/Saudi Arabia/6732-1/2007A/falcon/Saudi Arabia/6732-2/2007A/ostrich/Saudi Arabia/6732-3/2007A/chicken/Saudi Arabia/6732-4/2007	V33I	Chen *et al.* (2006)
